# miR-221 Promotes Epithelial-Mesenchymal Transition through Targeting PTEN and Forms a Positive Feedback Loop with β-catenin/c-Jun Signaling Pathway in Extra-Hepatic Cholangiocarcinoma

**DOI:** 10.1371/journal.pone.0141168

**Published:** 2015-10-26

**Authors:** Jianguo Li, Lei Yao, Guodong Li, Donglai Ma, Chen Sun, Shuang Gao, Ping Zhang, Feng Gao

**Affiliations:** 1 Department of General Surgery, the First Affiliated Hospital of JILIN University, Changchun, 130021, P.R. China; 2 Department of General Surgery, the Second Affiliated Hospital of Harbin Medical University, Harbin, 150086, P.R. China; 3 Department of General Surgery, the Fourth Affiliated Hospital of Harbin Medical University, Harbin, 150001, P.R. China; 4 Heilongjiang Nursing College, Harbin, 150086, P.R. China; China Medical University, TAIWAN

## Abstract

Extrahepatic cholangiocarcinoma (EHCC) is a refractory malignancy with poor prognosis due to its early invasion, metastasis and recurrence after operation. Therefore, understanding the mechanisms of invasion and metastasis is the key to the development of new and effective therapeutic strategies for EHCC. In the present study we demonstrated that miR-221 promoted EHCC invasion and metastasis through targeting PTEN and formed a positive feedback loop with β-catenin/c-Jun signaling pathway. We found miR-221 was upregulated in EHCC specimens and CC cell lines. Moreover, miR-221 was found strongly associated with the metastasis and prognosis of EHCC patients. The expression of PTEN was downregulated in EHCC patients and CC cell lines, and was further demonstrated as one of the downstream targets of miR-221. In addition, our data indicated that β-catenin activated miR-221 through c-jun, while miR-221 enhanced β-catenin signaling induced-epithelial-mesenchymal transition (EMT) by targeting PTEN, hence forming a positive feedback loop in EHCC cell lines. In conclusion, our results suggested that miR-221 promotes EMT through targeting PTEN and forms a positive feedback loop with β-catenin/c-Jun signaling pathway in EHCC.

## Introduction

Extrahepatic Cholangiocarcinoma (EHCC) is a malignant epithelial neoplasm with bile duct epithelial differentiation. The incidence and mortality of EHCC are rising worldwide. The intraductal and extraductal invasion, metastasis to regional lymph nodes and liver sites are the main prognostic factors in EHCC patients [1]. Moreover, local and distant recurrences occur in many EHCC patients after resection [2]. Therefore, it is necessary to elucidate the mechanisms of the invasion and metastasis of EHCC.

miRNAs are a class of short, endogenous, non-coding RNAs that can negatively regulate the expressions of various protein-coding genes by base pairing with their 3’ untranslated region (3’-UTR), leading to their post-transcriptional translation inhibition or mRNA degradation [3]. Recently, microRNAs are thought to play important roles in cancer treatment by regulating the expression of various oncogenes and tumor suppressors [4]. Among these, miR-221 is one of the earliest known oncogene and is commonly over expressed in various types of cancers [5–7]. To date, there is a general agreement about miR-221 that it is involved in migration and invasion of various types of cancers [8–9]. However, the function of miR-221 in EHCC is still not clear yet.

PTEN (phosphatase and tensin homolog deleted on chromosome 10) is one of the most commonly altered tumor suppressors in human cancers and a key regulator of cell growth and apoptosis [10]. Functionally, PTEN tightly regulates transcriptional activity or protein stability of β-catenin and in subsequent increase in protein synthesis, cell cycle progression, migration and invasion, especially epithelial-mesenchymal transition (EMT) [11–12]. Additionally, recent studies suggest that β-catenin-mediated transcription could be regulated by microRNAs, including miR-221/222 [5], which shed light on the precise regulation of Wnt/β-catenin signaling in EMT. Most importantly, recent report has identified that c-Jun, which is an important mediator of β-catenin functions, induced the expression of the oncomiRs miR-221/222 in prostate carcinoma and glioblastoma cells [13–14].

In the present study, miR-221 and PTEN expression levels were detected in EHCC tissues and CC cell lines, PTEN was demonstrated as a direct transcriptional target of miR-221 in EHCC cell lines. Relationships between miR-221 and clinical characteristics of EHCC patients were further examined. (Our result indicated miR-221 could mediate β-catenin/c-Jun-induced EMT by targeting PTEN in the progression of EHCC, and the activation of β-catenin/c-Jun signaling pathway further promoted the expression of miR-221 in EHCC.)

## Materials and Methods

### Patients and tissue samples

EHCC and para-carcinoma tissues were collected from 25 EHCC patients who underwent potentially curative surgery between 2010 and 2011 at the Second Affiliated Hospital of Harbin Medical University (Harbin, China) and were verified by a pathologist. The hard and firm tumor tissues were trimmed free of normal tissue and snap frozen in liquid nitrogen immediately after resection. No patient in the current study received chemotherapy or radiation therapy before the surgery. The tumor stage was classified according to the 7th tumor-node-metastasis classification of the International Union against Cancer (UICC). All the patients signed informed consent forms according to our institutional guidelines, and the study was approved by Institutional Review Board (IRB) protocols of Harbin Medical University. Information about gender, age, stage of disease, and histological factors was extracted from medical records.

### RNA extraction and quantitative real-time PCR (qRT-PCR)

qRT-PCR was used to confirm the expression levels of mRNAs and miRNAs. For mRNAs detection, total RNA from cultured cells and fresh surgical tissues was extracted using Trizol (Invitrogen, USA) according to the protocol. Reverse transcription was performed according to the protocol of High Capacity cDNA Reverse Transcription Kit (Applied Biosystems, USA). For miRNAs detection, total miRNA from cultured cells and fresh surgical tissues was extracted using the mirVana miRNA Isolation Kit (Ambion, USA), according to the manufacturer’s protocol. Complimentary DNA was synthesized from 2μg of total RNA using the High Capacity cDNA Reverse Transcription Kit (Applied Biosystems, USA). The expression levels of miRNA and mRNA were assessed with qRT-PCR using Power SYBR^®^ Green (Applied Biosystems, USA) by an Applied Biosystems 7500 Sequence Detection system. The expression levels of mRNA and miRNA were defined based on the threshold cycle (Ct), and the relative expression levels were calculated by using the 2^-ΔΔCt^ method, with the expression levels of β-actin mRNA and U6 small nuclear RNA as reference. The names of the genes and the primers are listed in S1 Table.

### Cell culture and quick transfection

The human EHCC cell lines QBC 939 and HuCCT 1 used in this study were purchased from American Type Culture Collection (Manassas, USA). The human IHCC cell line RBE and HCCC 9810 were obtained from the Cell Bank of the Chinese Academy of Sciences (China). HiBECs was purchased from PriCells Biomedical Technology Co., Ltd. (China). All the cells were cultured according to the manufacturer’s instructions.

The hsa-miR-221 mimic, negative control (NC) oligonucleotides, the has-miR-221 inhibitor (antagomirs) and scramble oligonucleotides were purchased from GenePharm (GenePharm, China). Both of the QBC 939 and HuCCT 1 cells were infected with the miR-221 inhibitor or scramble oligonucleotides (100 nmol/L) using Lipofectamine 2000 (Invitrogen, USA). After 24 hours, the cells were collected for qRT-PCR or Western blot analysis.

The specific PTEN siRNA (50 nM), c-Jun siRNA (50 nM) and their non-specific scramble siRNA (NC-siRNA, 50 nM) were purchased from Stanta Cruz (Santa Cruz Biotechnology, USA) and the transfection of siRNAs was performed for 48 hours with a Lipofectamine 2000 transfection kit (Invitrogen, USA) according to manufacturer’s recommended protocol. The efficiency of siRNA on knocking down PTEN or c-Jun was examined by western blotting analysis.

### Drugs and reagents

6-bromoindirubin-3`-oxime (BIO) was purchased from Wako (Japan). Stock solution of BIO (1μM) was prepared in Dimethy sulphoxide (DMSO) and stored in aliquots in −20°C freezers in a dark sealed container.

### Construction of Vector construction and luciferase reporter assay

For the luciferase assays, the potential miR-221 binding site in the PTEN 3`-UTR was predicted using Target Scan (www.targetscan.org) and miRanda (www.microRNA.org). The 3’-UTR of the PTEN mRNA and a mutant PTEN mRNA were synthesized and cloned into the Xba I site of a pGL3 basic vector (Promega, United States) downstream from the luciferase stop codon and were designated as pGL3-wt-PTEN and pGL3-mt-PTEN respectively. Then, both of the QBC 939 and HuCCT 1 cells (1 × 10^5^ cells/well) cultured in 24-well plates were co-transfected with the pGL3-Control (0.4mg), pGL3-wt-PTEN (0.4mg) or pGL3-mt-PTEN (0.4mg) plasmid, the pRL-TK luciferase reporters (25ng/well) and pcDNA-miR-221 (20nmol/L) or pcDNA-miR-NC (20nmol/L) using Lipofectamine 2000 (Invitrogen, USA). After 48 hours, the cells were harvested and luciferase activities were measured using a Dual-Luciferase Reporter Assay kit (Promega, USA).

### Western Blot

Protein lysates were separated using desired SDS-PAGE gel electrophoresis and transferred to nitrocellulose membranes (Amersham Pharmacia Biotech, USA). The membrane was probed with the following antibodies: anti-PTEN, anti-β-catenin, anti-c-Jun, anti-MMP2, anti-E-cadherin and anti-N-cadherin (Santa Cruz Biotechnology, USA). Finally, the membrane was probed with Alexa Fluor^®^ 680 donkey anti-mouse IgG (H+L) (Invitrogen). Antibody binding was detected by Odyssey^™^ Infrared Imaging System (Li-Cor, Lincoln, NE). The names of the antibodies are listed in S2 Table.

### Cell migration and invasion assays

The invasive potential of cells was measured in 6.5 micrometers Transwell with 8.0 micrometers Pore Polycarbonate Membrane Insert (Corning, USA) according to the manufacturer’s instructions. The filter of top chamber was matrigel-coated with 50μl of diluted matrigel following the standard procedure and incubated at 37°C for 2h. The lower chambers were filled with 600μl of DMEM medium containing 5% FBS as chemoattractant for a further 24h [2]. Cells were serum-free-starved overnight, harvested and resuspended in migration medium (DMEM medium with 0.5% BSA). Then the suspension of 5,000 cells in 100μl migration medium was added into each top chamber. After the cells were incubated for 16h, the non-invading cells that remained on the upper surface were removed with a cotton swab. The invasive cells on the lower surface of the membrane insert were fixed with 4% paraformaldehyde for 30min, permeabilized with 0.2% Triton X-100 at room temperature for 15min, and then stained with 0.1% crystal violet for 5min. The number of cells on the lower surface, which had invaded through the membrane, was counted under a light microscope in five random fields at a magnification of 100X. The procedure for transwell migration assays was the same as the transwell invasion assay except that the filter of top chamber was not coated with matrigel. The experiments were repeated 3 times independently and results were given as means ± SD.

### Statistical analysis

All the presented data were expressed as the mean ± SD and representative results were from at least three independent experiments. Statistical comparisons were calculated by Student’s two-tailed t-test. When multiple comparisons were possible, ANOVA coupled with Tukey correction was used. Cox regression analysis by backward variable selection was performed to analyze all factors in the Table 1. Correlation analysis between relative expressions of PTEN and miR-221 was examined by Spearman p test. P<0.05 was considered statistically significant. Statistical analysis was carried out using SPSS 21 (IBM Corporation Software Group, USA) or the GraphPad Prism 5.0 software package (GraphPad Software, Inc., USA).

**Table 1:** Relationship between miR-221 expression and clinicopathological features in EHCC patients.

Clinicopathological features	n	miR-221		P value
		**low**	**high**	
Age (yr)				0.695
<60	13	8	5	
≥60	12	6	6	
Gender				0.227
Male	14	6	8	
Female	11	8	3	
Tumor size (cm)				0.428
<2	14	8	6	
≥2	11	4	7	
Pathological type				1.000
Adenocarcinoma	23	13	10	
Mucocellulare carcinoma	0	0	0	
Adenosquamous carcinoma	2	1	1	
Squamous carcinoma	0	0	0	
Undifferentiated carcinoma	0	0	0	
Cell differentiation				0.672
Well	4	3	1	
Moderately	7	4	3	
Poorly	14	7	7	
Bismuth classification				0.407
Bismuth I	7	5	2	
Bismuth II	12	8	4	
Bismuth III	5	2	3	
Bismuth IV	1	0	1	
Lymphatic node metastasis				0.042
Absent	14	7	7	
Present	11	10	1	
Clinical stages				0.033
I+II	14	8	6	
III+IV	11	1	10	

P<0.05 is significant.

## Result

### Overexpression of miR-221 and the association with the clinicopathological significance in human EHCC tissues

**Fig 1:**
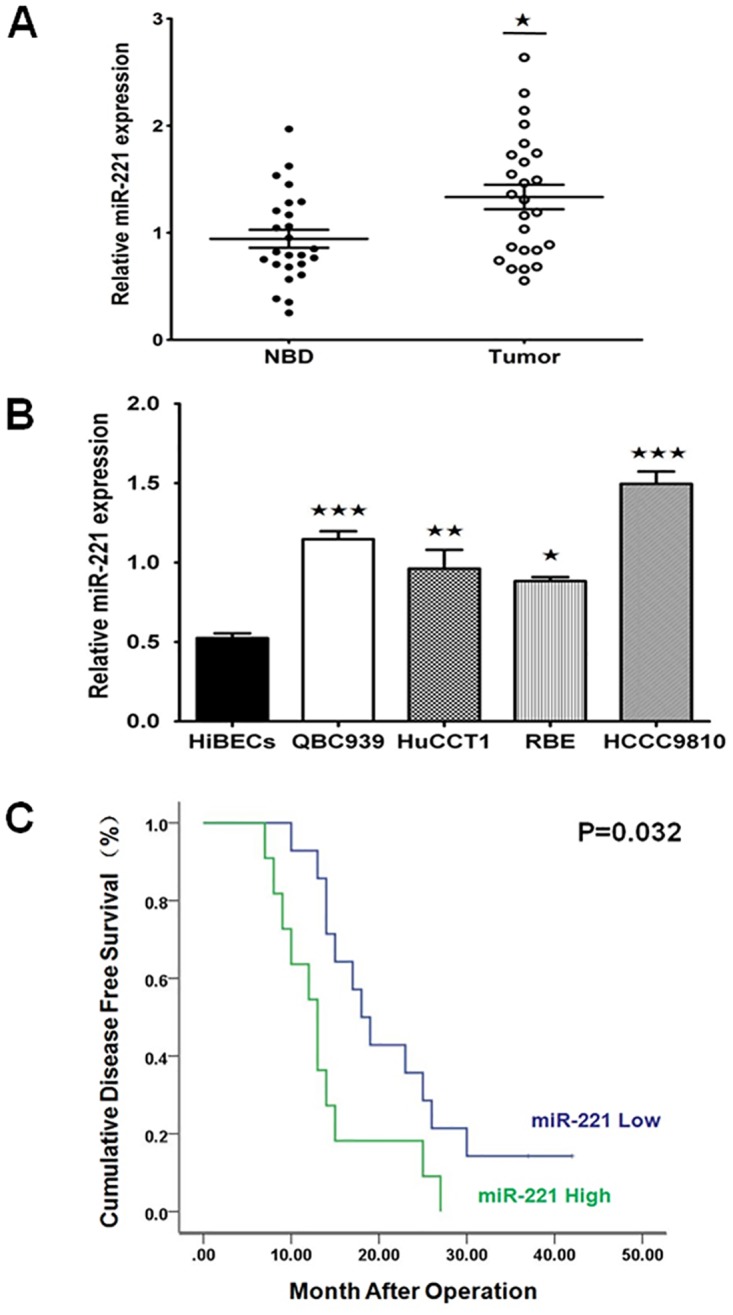
miR-221 expression in human EHCC tissues and CC cell lines, and the relationship between miR-221 expression and disease-free survival in EHCC patients. (A) The relative expression of miR-221 in 25 primary EHCC tissues compared to their matched normal adjacent bile duct tissues determined by qRT-PCR. U6 was used as the internal control (^★^P<0.05). (B) The relative expression of miR-221 in CC cell lines (QBC939, HuCCT1, RBE and HCCC9810) compared to the HiBECs determined by qRT-PCR. U6 was used as the internal control (^★^P<0.05, ^★★★^P<0.001). (C) miR-221 predicts disease-free survival in EHCC patients. The Kaplan-Meier curve of disease-free survival in patients with low miR-221 expression (n = 14) and high miR-221 expression (n = 11). The median disease-free survival time was 22.00 months and 13.91 months in low- and high- miR-221 group respectively (P = 0.032).

To investigate the biological role of miR-221 in human EHCC development, we analyzed the expression of miR-221 in human EHCC tissues and their paired para-carcinoma tissues by qRT-PCR. Compared with the para-carcinoma tissues, the relative expression level of miR-221 in EHCC tissues was significantly higher (P<0.05, Fig 1A). To further characterise miR-221 expression in CC cell lines, HiBECs, EHCC cell lines QBC939, HuCCT1 and IHCC cell lines RBE, HCCC9810 were examined. qRT-PCR analysis revealed that the expression level of miR-221 was significantly higher in all of the CC cell lines compared with the expression levels in HiBECs (P<0.05, Fig 1B). These results suggest that up-regulation of miR-221 may be, at least in part, involved in most of human CC development. Then we further evaluated the clinical value of miR-221 in EHCC patients who were divided into two cohorts according to the median value (1.334) of the expression level of miR-221. The correlation between miR-221 and clinicopathological characteristics was then analyzed. Weobserved that miR-221 showed lower expression levels in specimens with lymphatic metastasis (P = 0.042, Table 1) and the advanced clinical stages (stage III, IV vs I, II) (P = 0.033, Table 1). However, no association of miR-221 was observed with age, gender, tumor size, different pathological types, cell differentiation and Bismuth classification (P>0.05, Table 1). Then multivariable Cox’ proportional hazards model was performed to analyze all factors in the Table 1. The result indicated that only miR-221 was selected into the model (P<0.05). So we concluded that miR-221 was an independent prognostic indicator in EHCC. Kaplan-Meier analysis showed that up-regulation of miR-221 was correlated with decreased disease-free survival (Fig 1C, P = 0.032).

### The expression of PTEN, β-catenin, c-jun and miR-221 and PTEN expression levels are inversely regulated in EHCC tissues

**Fig 2:**
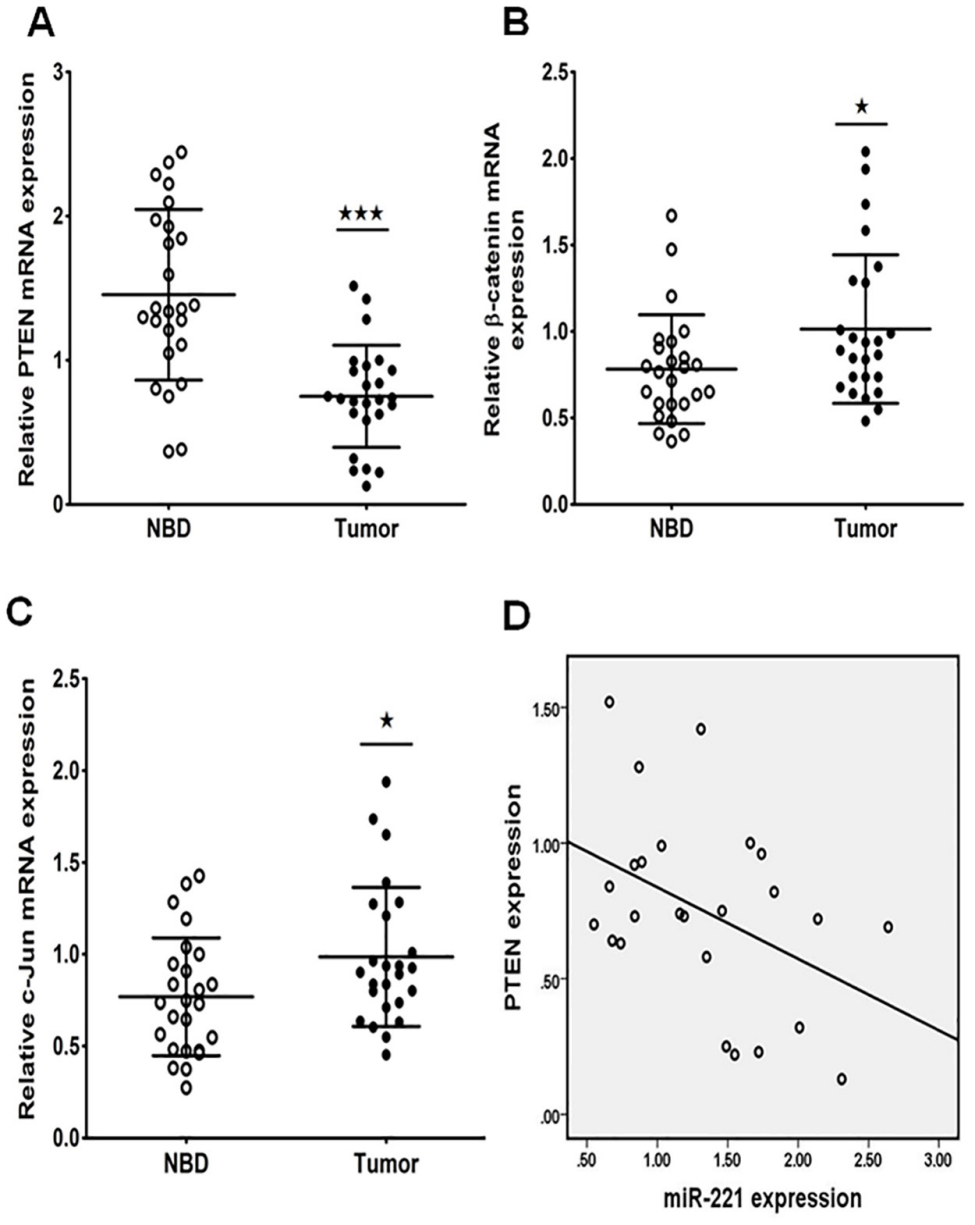
The relative expression of PTEN, β-catenin and c-Jun in EHCC and their paired para-carcinoma tissues. (A, B, C) The mRNA expression level of PTEN, β-catenin and c-Jun in the primary EHCC and their paired para-carcinoma tissues determined by qRT-PCR. β-actin was used as the internal control (^★^P<0.05, ^★★★^P<0.001). (D) The inverse correlation of PTEN and miR-221 expression levels was examined by Spearman correlation analysis (R = -0.429, P = 0.032).

In order to determine the clinical significance target genes of miR-221 in EHCC, we used Sanger miRNA database (http://www.mirbase.org/) and Target scan (http://www.targetscan.org/) to predict the candidates of miR-221. The result indicated that PTEN might be one of the potential targets of miR-221. Moreover, it has been reported that miR-221/222 regulated TRAIL-resistance and enhanced tumorigenicity through PTEN down-regulation in human aggressive non small cell lung cancer and hepatocarcinoma cells [9]. Thus, we examined PTEN and β-catenin, c-jun expression in the EHCC specimens. It was found that the mRNA level of PTEN was pronouncedly decreased while β-catenin and c-jun was upregulated in EHCC tissues compared to their paired para-carcinoma tissues (P<0.05, Fig 2A, 2B and 2C). Then the Spearman correlation analysis was applied to compare the relative expression levels of PTEN and miR-221 in the EHCC specimens. We obtained a statistically significant inverse correlation (R = -0.429, P = 0.032) in a total of 25 EHCC tissues (Fig 2D). These data suggested that miR-221 expression was inversely correlated with PTEN and miR-221 might play a critical role on PTEN expression in EHCC patients.

### miR-221 directly targets PTEN in EHCC cells

**Fig 3:**
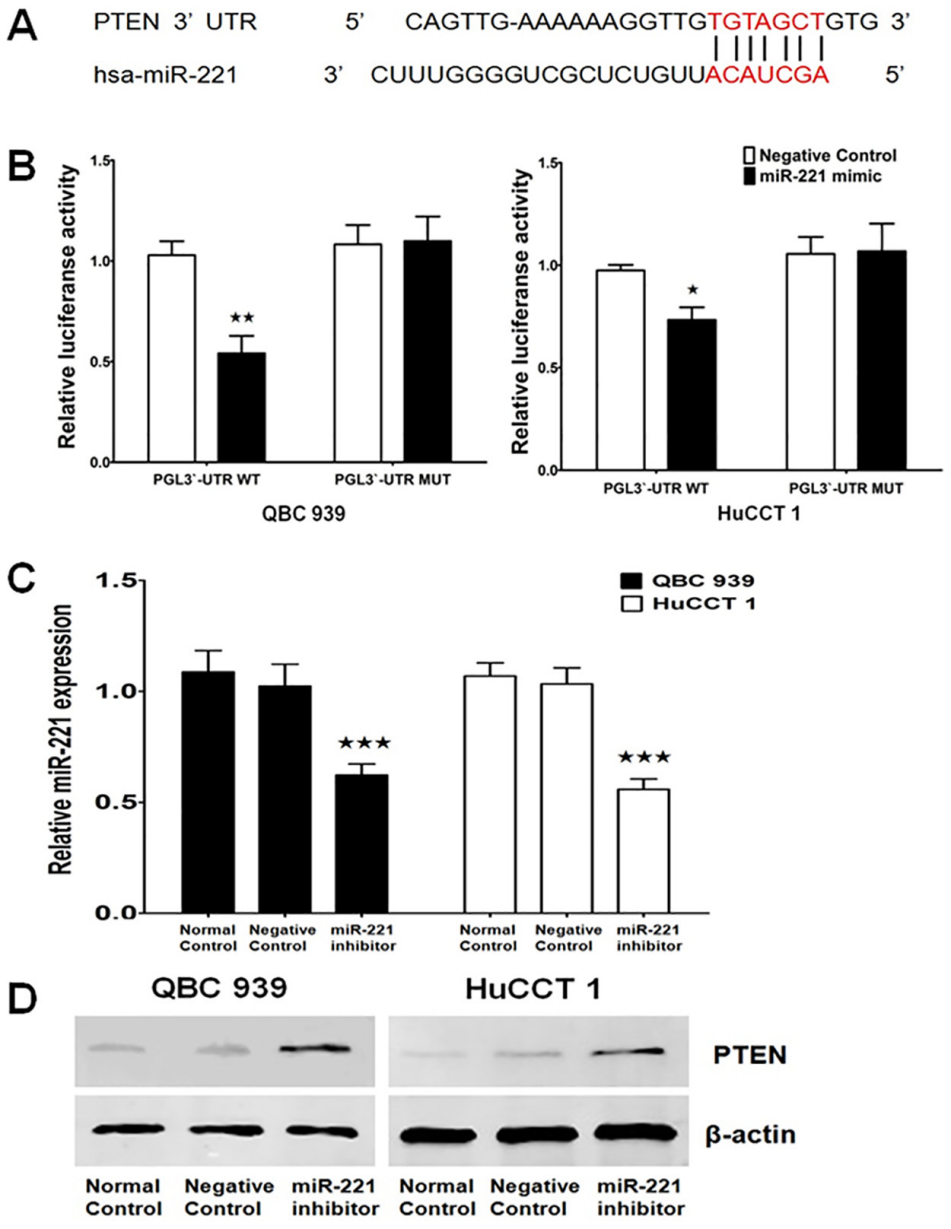
PTEN is a direct miR-221 target. (A) Putative miR-221 binding sequences in the 3’-UTR of PTEN mRNA. (B) 3’-UTR luciferase reporter assay showed a reduction of relative luciferase activity of wild-type PTEN 3’-UTR by pre-miR-221 in QBC939 and HuCCT1 cells (^★^P<0.05). (C) qRT-PCR analysis of expression of miR-221 treated with miR-221 inhibitor in QBC939 and HuCCT1 cells (^★★★^P<0.001). U6 was used as an internal control. (D) Western blot analysis of PTEN expression transfected with miR-221 inhibitor in QBC939 and HuCCT1 cells. β-actin expression levels were used as internal loading control.

To investigate the underlying mechanism of miR-221 in EHCC, TargetScan (www.targetscan.org) and miRanda (www.microRNA.org) were used to search for potential targets of miR-221. The result showed that the 3’-UTR of PTEN contained the complementary site for the seed region of miR-221 (Fig 3A). To verify that PTEN is one of the direct targets of miR-221, we constructed a reporter vector consisting of the luciferase coding sequence followed by the 3’-UTR of PTEN. A dual luciferase reporter assay was performed in both QBC 939 and HuCCT 1 cell line. As shown in Fig 3B, miR-221 could significantly decrease the luciferase activity of the wild type PTEN 3’-UTR compared with the vector-only control. Additionally, partial mutation of the perfectly complementary sites in the 3’-UTR of PTEN abolished the suppressive effect due to the disruption of the interaction between miR-221 and PTEN.

Then miR-221 inhibitor oligonucleotides were transfected into the EHCC cell lines to decrease the endogenous level of miR-221 to further investigate the biological function of miR-221 in QBC939 and HuCCT1 cells (Fig 3C). As shown in Fig 3D, PTEN protein expression was increased by transfecting with the miR-221 inhibitor compared to the control groups in both QBC939 and HuCCT1 cells. These data suggest that PTEN expression was primarily inhibited by miR-221 and PTEN was a direct target of miR-221 in EHCC cell lines.

### PTEN was involved in miR-221-regulated EMT in EHCC cell lines

**Fig 4:**
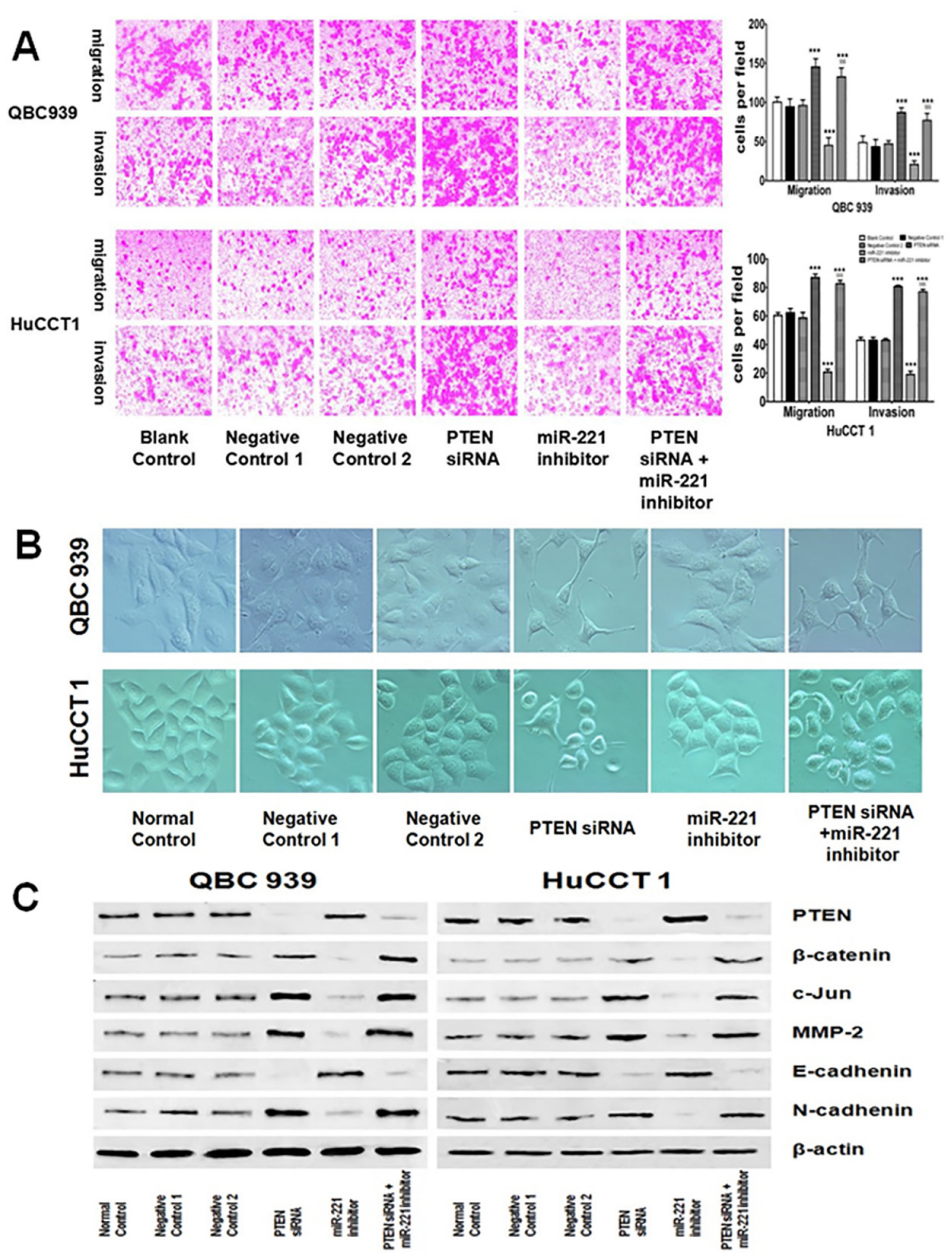
PTEN is involved in miR-221 regulated EMT through β-catenin/cJun signaling pathway induced migration and invasion. (A) Both QBC939 and HuCCT1 cells were transfected with PTEN siRNAs then treated with or without miR-221 inhibitor to evaluate cell invasion and migration activities using a cell invasion assay in transwell chambers. Representative images of cells migrated into the lower chamber were shown (left panel, original magnification: X100), and quantitative data were also presented (right panel). (right panel, ^★★★^P<0.001 indicates PTEN siRNAs, miR-221 inhibitor or a mixed PTEN siRNAs with miR-221 inhibitor *vs*. BC. ^$$$^P<0.001 means a mixed PTEN siRNAs with miR-221 inhibitor *vs*. miR-221 inhibitor). The average numbers of cells per field of view from three different experiments are plotted. Negative control 1: mock control for siRNA containing only transfection reagent; Negative control 2: negative control for miR-221 inhibitor. (B) Morphological investigations of QBC939 and HuCCT1 cells. The cells were transfected with PTEN siRNAs then treated with or without miR-221 inhibitor. Cells transfected with siRNA containing only transfection reagent or scrambled oligonucleotide were used and named as Negative control 1 and Negative control 2 respectively (original magnification: ×200). (C) Both of the QBC939 and HuCCT1 cells were transfected with siRNA containing only transfection reagent, scrambled oligonucleotide, PTEN siRNAs and treated with or without miR-221 inhibitor and then the cells lysate were examined with indicated antibodies by Western blot. β-actin was used as loading control.

To investigate whether PTEN was involved in miR-221-regulated migration and invasion in EHCC cell lines, both QBC 939 and HuCCT 1 cells were transfected with PTEN siRNAs and treated with or without miR-221 inhibitor respectively. Then transwell migration and invasion assays were performed. It was found that transfection with miR-221 inhibitor significantly suppressed cell migration and invasion capacity in both QBC939 and HuCCT1 cells. When transfected with the PTEN siRNAs, cell migration and invasion capacity were significantly induced in both of the cell lines. However, these inductions were inhibited by the treatment with miR-221 inhibitor in combination with PTEN siRNA (Fig 4A). Fig 4B depicted the dramatic morphological change associated with down-regulation of miR-221 repressed the EMT by targeting PTEN in both QBC939 and HuCCT1 cells. After the EHCC cells were transfected with PTEN siRNAs, the cells displayed a spindle-shaped morphology, the cell-to-cell adhesions became weak and the cells were scattered. However, the miR-221 inhibitor-treated cells developed a cobblestone-like morphology and cell-to-cell adhesion was more intact compared with the control groups. Interestingly, the cells treated with both PTEN siRNAs and miR-221 inhibitor resembled closely to the PTEN siRNAs cells. Then western blot analysis was performed to further investigate whether PTEN was involved in miR-221-regulated migration and invasion in both QBC939 and HuCCT1 cells. We found that PTEN and E-cadherin protein expression level was downregulated while β-catenin, cJUN, MMP-2 and N-cadherin protein expression levels were upregulated when transfected with the PTEN siRNAs, but results were only obtained when treated with miR-221 inhibitor, and these results were abrogated when treated with miR-221 inhibitor in combination with PTEN siRNA compared with being treated with single miR-221 inhibitor in both QBC939 and HuCCT1 cells (Fig 4C). These data suggest that PTEN was involved in repressing miR-221-mediated EMT in EHCC cells.

### miR-221 downregulation inhibits migration and invasion through β-catenin-signaling-pathway-induced EMT in EHCC cell lines

**Fig 5:**
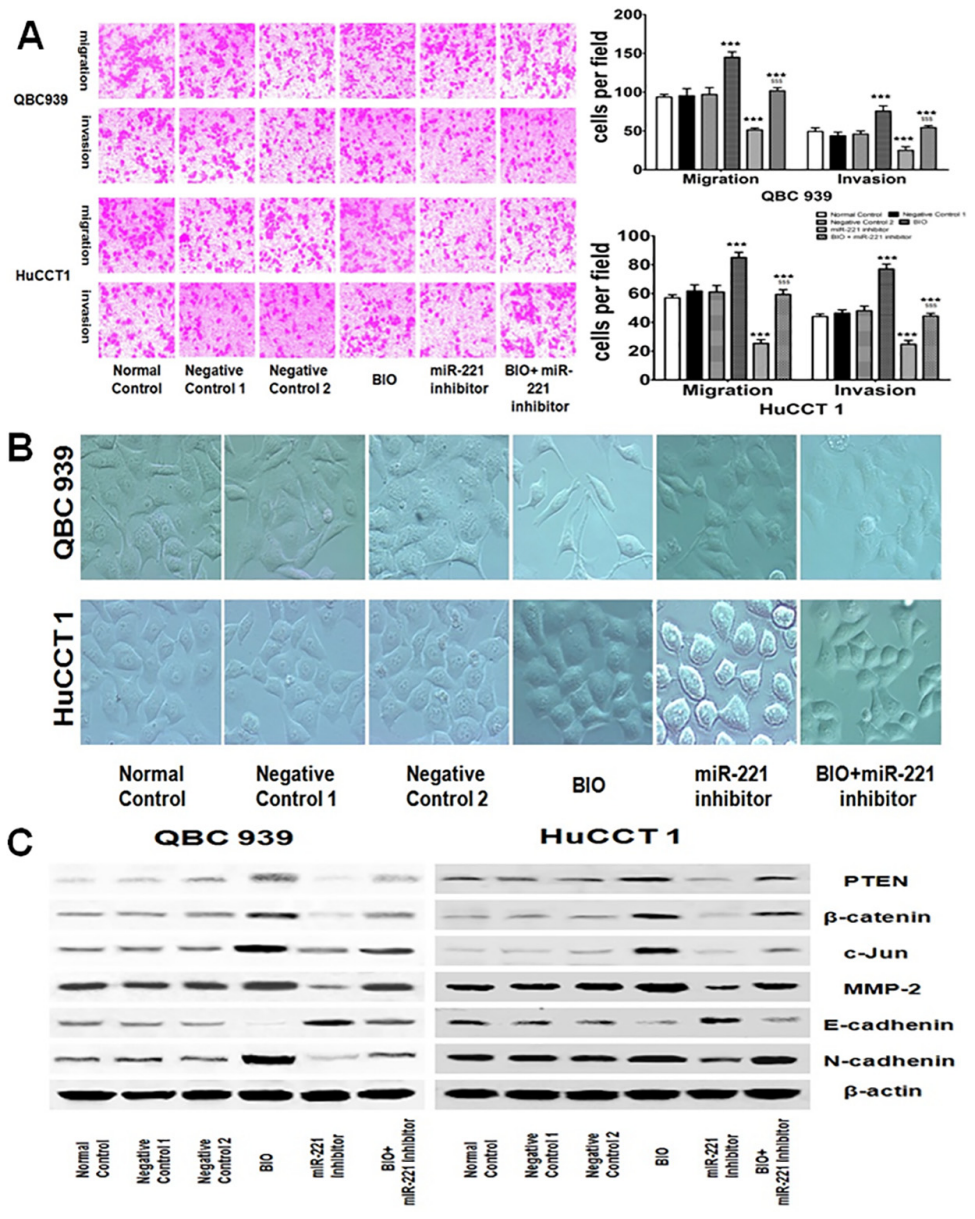
miR-221 downregulation inhibits migration and invasion through β-catenin signaling pathway induced EMT in EHCC cell lines. (A) Both QBC939 and HuCCT1 cells were treated with BIO and combination with or without miR-221 inhibitor to evaluate cell invasion and migration activities by transwell chambers. Representative images of cells migrated into the lower chamber were shown (left panel, original magnification: X100), and quantitative data were also presented (right panel). (right panel, ^★★★^P<0.001 indicates BIO, miR-221 inhibitor or a mixed BIO with miR-221 inhibitor *vs*. NC. ^$$$^P<0.001 means a mixed BIO with miR-221 inhibitor *vs*. miR-221 inhibitor). The average numbers of cells per field of view from three different experiments are plotted. (B) Representative images of morphological investigations of QBC939 and HuCCT1 cells (original magnification: ×200). (C) Both of the QBC939 and HuCCT1 cells were treated with BIO and treated with or without miR-221 inhibitor and then the cells lysate were examined with indicated antibodies by Western blot. β-actin was used as loading control. Negative control 1: negative control for BIO with only 0.1% DMSO; Negative control 2: negative control for miR-221 inhibitor.

As PTEN could induce the nuclear translocation of β-catenin in carciongenesis [15], so we hypothesized that the inhibition of β-catenin signaling pathway by miR-221 inhibitor may repress EMT and migration/invasion in EHCC. It has been well established that activation of the canonical Wnt pathway inhibits GSK-3β from phosphorylating β-catenin, resulting in the accumulation of active β-catenin polypeptide species that translocate into the nucleus. Small molecule inhibitors of GSK-3β, BIO, have been shown to be effective at stabilization and activation of β-catenin [16]. Thus, both QBC 939 and HuCCT 1 cells were treated with BIO and with or without miR-221 inhibitor respectively. Then transwell migration and invasion assays were performed. It was found that transfection with miR-221 inhibitor significantly suppressed cell migration and invasion capacity in both QBC939 and HuCCT1 cells. When treated with the BIO, cell migration and invasion capacity were significantly promoted in both of the cell lines. However, these inductions were inhibited by the treatment with miR-221 inhibitor in combination with BIO (Fig 5A). As shown in Fig 5B, treatment with BIO in both of the cell lines induced a morphological change from cobblestone-shaped epithelial-like form to a spindle-shaped mesenchymal form, the cell-to-cell adhesions became weak and the cells were scattered. By contrast, inhibition of miR-221 in QBC939 and HuCCT1 cells led to more cobblestone-shaped epithelial-like characteristics. However, the cells treated with both BIO and miR-221 inhibitor resembled closely to the BIO-alone-treated cells. Moreover, the expression of a set of EMT-related protein markers was also altered along with the morphological changes. PTEN and E-cadherin protein expression level was downregulated while β-catenin, cJUN, MMP-2, N-cadherin protein expression levels were upregulated when treated with the BIO, but results were obtained when treated with miR-221 inhibitor, and these results were abrogated when treated with BIO in combination with miR-221 inhibitor compared with being treated with single miR-221 inhibitor in both QBC939 and HuCCT1 cells (Fig 5C). These data suggested that miR-221 downregulation inhibits migration and invasion through β-catenin signaling pathway induced EMT in EHCC cell lines.

### β-catenin/c-Jun signaling pathway induces the expression of the oncogenic miR-221 in EHCC

**Fig 6:**
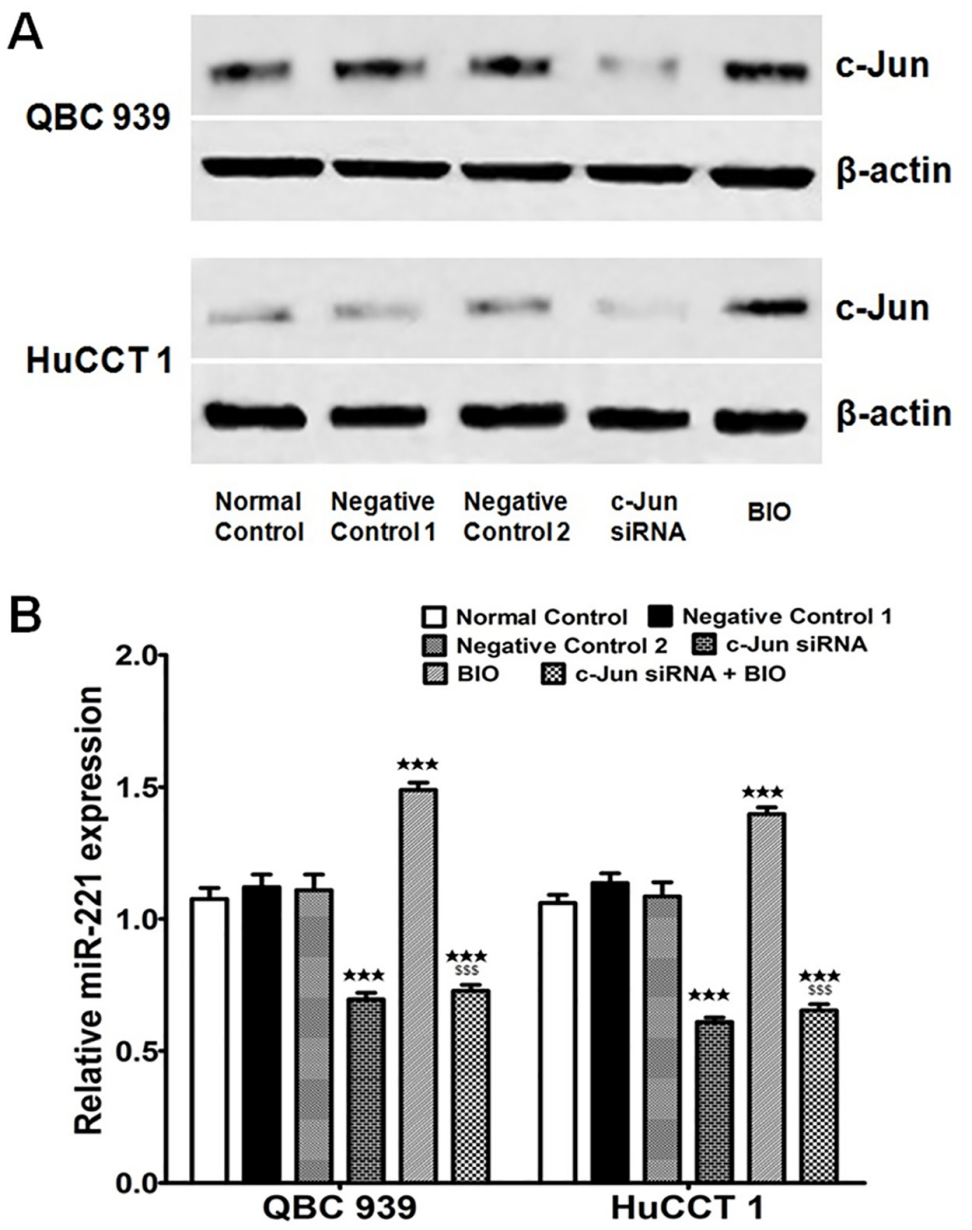
β-catenin/c-Jun signaling pathway promotes miR-221 expression. (A) PTEN protein level with c-Jun siRNA or BIO treatments detected by western blot. Antibodies include: PTEN and β-actin. Negative control 1: mock control for siRNA containing only transfection reagent; Negative control 2: mock control for BIO with only 0.1% DMSO. (B) Both of the QBC939 and HuCCT1 cells were transfected c-Jun siRNAs and treated with or without BIO for desired time. Then the relative expression of miR-221 was determined by qRT-PCR. U6 was used as the internal control. (^★★★^P<0.001 indicates c-Jun siRNAs, BIO or a mixed c-Jun siRNAs with BIO *vs*. NC. ^$$$^P<0.001 means a mixed c-Jun siRNAs with BIO *vs*. BIO)

It has been reported NF-kB and c-Jun induce the expression of miR-221 and miR-222 in prostate carcinoma and glioblastoma cells [14]. Additionally, c-Jun was a downstream target of β-catenin, so activation of β-catenin/c-Jun signaling pathway may induce the expression of miR-221 in EHCC. Thus, we examined the effects of c-Jun on the expression of the oncogenic miR-221 in both QBC939 and HuCCT1 cells. The cells were transfected with c-Jun siRNAs or scramble oligos and simultaneously treated with or without 1 μm 6-bromoindirubin-39-oxime (BIO) (sigma, USA) for 24 hours, which is an inhibitor of glycogen synthase kinase 3 (GSK3), with the function of activating β-catenin signal pathway. Western blot analysis showed that transfection of c-Jun siRNAs decreased the expression of c-Jun protein levels compared with the control group. BIO could significantly increase c-Jun protein expression (Fig 6A). Then the expression of miR-221 levels was further examined by qRT-PCR. After c-Jun siRNAs transfection, miR-221 expression levels were shown as in Fig 6B. Results were obtained when the cells were treated with BIO in both QBC939 and HuCCT1 cells. Moreover, when treated with c-Jun siRNAs and BIO simultaneously, there was no significant changed on miR-221 expression compared with being transfected with c-Jun siRNAs alone. These results demonstrated that β-catenin/c-Jun signaling pathway was involved in miR-221 expression regulation in EHCC.

## Discussion

Extrahepatic CC is a malignant epithelial neoplasm with bile duct epithelial differentiation. It often invades in the lateral direction as intraductal invasion and in the vertical direction as extraductal invasion [17]. Extrahepatic CC also metastasizes to regional lymph nodes and liver sites, which are the main prognostic factors in EHCC patients. Thus it is necessary to elucidate the mechanisms of invasion and metastasis of EHCC. Recent reports have demonstrated microRNAs as an important regulator of tumor progression and metastasis [18]. In EHCC, many miRNAs have been proved acting as a regulator to known genes that are involved in the pathology of tumorigenesis and metastasis. miR-221 was found to be associated with the process of metastasis in many human cancers including breast, lung, liver, prostate and bladder [5–9]. Concerning the relationship between miR-221 expression and clinicopathological features, in the present study we found that miR-221 expression was significantly increased in human EHCC tissues and CC cell lines, and miR-221 expression level was associated with risk of relapse and metastasis. PTEN was further demonstrated as one of the targets of miR-221, which is partly consistent with Garofalo et al [9]. We also found PTEN was involved in miR-221-regulated EMT through β-catenin/cJun signaling pathway in EHCC cell lines. Additionally, our results further implicated that β-catenin/c-Jun signaling pathway induced the expression of miR-221 in EHCC. Thus, miR-221 and β-catenin/c-Jun signaling pathway form a positive feedback loop via PTEN to promote EMT and metastasis.

Previous studies have provided evidence for the important anti-oncogene by regulating different downstream targets roles of deregulated expression of miR-221 in various types of cancers and miR-221 has been found to be associated with the process of metastasis in many human cancers [5–9]. Moreover, recent reports have shown that PTEN upregulated at both the mRNA and protein levels in several types of human cancers [10]. Additionally, recent reports demonstrated that miR-221/222 regulated TRAIL-resistance and enhanced tumorigenicity through PTEN and TIMP3 down-regulation in aggressive non small cell lung cancer and hepatocarcinoma cells [9]. However, how miR-221 expression is regulated and its role in EHCC migration and invasion has yet to be elucidated. Our present study showed that the expression of miR-221 was up-regulated in EHCC tissues. The clinical analysis indicated that up-regulation of miR-221 was correlated with lymphatic metastasis and advanced clinical stages. These data suggested that miR-221 might be associated with the invasion and metastasis in EHCC. Recent studies have demonstrated that miR-221 suppressed H460 cells migration by directly targeting PTEN and TIMP3 [9]. This observation is in accordance with our finding of miR-221 upregulation and targeting PTEN to suppress EHCC cells migration and invasion.

The mechanisms of miR-221 regulating cell growth, metastasis and inducing invasion could be correlated to different networks between miR-221 and other target genes. We applied bioinformatic methods and predicted the tumor suppressor PTEN might be one of the potential targets of miR-221. Further investigation showed that miR-221 suppressed the activity of a luciferase reporter gene fused with the 3’-UTR of PTEN mRNA, which is dependent on the miR-221 binding sequence. Our data revealed that miR-221 directly targeted the 3’-UTR of PTEN, and that ectopic expression of miR-221 repressed the expression of PTEN protein level in EHCC cell lines.

Epithelial-to-mesenchymal transition (EMT) was first recognised as a central differentiation process in early embryogenic morphogenesis [19]. Numerous reports have supported that EMT has been implicated in cancer progression and metastasis. The loss of Epithelial cadherin (E-cadherin) expression and gain of Neural cadherin (N-cadherin) expression in cancer cells, sometimes called “the cadherin switch”, have functional significance in cancer progression and EMT [2]. Several oncogenic pathways such as TGF-β, Wnt and Notch signaling pathways have been shown to induce EMT [20]. More importantly, it has been firmly established that PTEN-induced activation of β-catenin/c-Jun signaling pathways has been shown to play a crucial role during the induction of EMT [21]. Moreover, microRNAs have recently emerged as potent regulators of EMT due to their ability to target multiple components involved in epithelial integrity or mesenchymal traits [22]. Thus, our data showed that the inhibition of miR-221 decreased the expression of β-catenin and its downstream targets c-Jun as well as MMP2, which is associated with invasion of tumors, and the expression of N-cadherin was downregulated, E-cadherin was upregulated. The next question therefore was whether simultaneous inhibition of PTEN by siRNAs together with miR-221 inhibitor would result in additive biological effects. Compared withusing miR-221 inhibitor alone, the addition of miR-221 inhibitor to PTEN siRNAs led to an impaired effect on cell migration and invasion. Like the expression of PTEN, E-cadherin was downregulated and β-catenin, c-Jun, MMP2, N-cadherin was upregulated.Thus, our data showed that inhibition of miR-221 could antagonize PTEN-mediated invasion and metastasis through regulation of β-catenin/c-Jun pathway in EHCC.

Recently, a number of papers have reported microRNAs are predominantly transcribed by RNA Polymerase II and own many of the common features typical of RNA Pol II genes, such as the regulation by core promoters and distal enhancers, or the capping and the polyadenilation of primary transcripts [14, 23–24]. Serval reports have been published describing transcription factors in either physiological or pathological contexts that regulate miRNA expression by binding to promoter regions [14, 25–26]. Silvia A. Ciafre et al have recently described the role of enhancers in the specific induction of miRNA expression [14]. In the present study, we demonstrated that c-Jun was strongly involved in miR-221/222 transcription enhancement in EHCC cell lines, as indicated by the great reduction of miR-221 expression in cells when depleted c-Jun via siRNAs. On the other hand, a notably stronger enhancing effect of miR-221 expression was demonstrated for BIO treatment. Interestingly, when treated with c-Jun siRNAs and BIO simultaneously, the enhancing effect of miR-221 expression was vanished compared with being treated with BIO alone. These results demonstrated that β-catenin/c-Jun signaling pathway was involved in promoting miR-221 expression in EHCC. Thus, our data indicated that miR-221 formed a positive feedback loop with β-catenin/c-Jun signaling pathway. This finding is partly consistent with the outcome of Bu P et al, which is increasing appreciation of microRNAs form regulatory motifs with protein regulators to confer robustness to biological processes and their subversion can expose cells to elevated risk of malfunction [27]. As we known, positive feedback could amplify a response and commit into a self-sustained mode that is autonomous to the original stimuli. Therefore we speculated that once induced by β-catenin/c-Jun signaling pathway, the miR-221-mediated feedback loop would allow EHCC cells to become more autonomous. This would promote the activation of EMT and enhance the ability of EHCC cells to invade and metastasize to new microenvironments, which would explain the strong pro-metastasis phenotype we have observed.

## Conclusion

In summary, our results have identified miR-221 as an onco-miRNA in human EHCC, which acted at least in part through the repression of PTEN. Increased expression of miR-221 in EHCC patients was correlated with lymphatic metastasis, advanced clinical stages and disease-free survival rate. Inhibition of miR-221 expression led to an induction of PTEN and activation of β-catenin/c-Jun signaling pathway, which accelerated EHCC cells invasion and migration through activation of EMT. Moreover, the activation of β-catenin/c-Jun signaling pathway got the function of promoting miR-221 expression in EHCC cells. In conclusion, this context suggested that miR-221 promoted EMT through targeting PTEN and formed a positive feedback loop with β-catenin/c-Jun signaling pathway in EHCC.

## Supporting Information

S1 Table Primers used for qRT-PCR. (DOC)

S2 Table Antibodies used for Western Blot. (DOC)

## Abbreviations

CC: Cholangiocarcinoma
EHCC: extrahepatic CC
PTEN: phosphatase and tensin homolog deleted on chromosome 10
NBD: normal bile duct
miRNA: microRNA
UTR: 3’-untranslated region
EMT: epithelial-mesenchymal transition
BIO: 6-bromoindirubin-3`-oxime
DMSO: Dimethy sulphoxide
GSK3: glycogen synthase kinase 3
qRT-PCR: quantitative real time Reverse Transcription-PCR
DMEM: Dulbelcco’s Modified Eagle Media
FBS: fetal bovine serum
UICC: International Union against Cancer
PBS: Phosphate buffer saline
IRB: Institutional Review Board
