# Supplementary material for: miR-221 Promotes Epithelial-Mesenchymal Transition through Targeting PTEN and Forms a Positive Feedback Loop with β-catenin/c-Jun Signaling Pathway in Extra-Hepatic Cholangiocarcinoma
Source: PLoS One. 2015 Oct 26;10(10):e0141168. doi: 10.1371/journal.pone.0141168 (PMC4621024; doi:10.1371/journal.pone.0141168)
Supplement: S1 Table — (DOC) [file pone.0141168.s001.doc]

| **Primers used for qRT-PCR** | |
| --- | --- |
| **Primer Name** | **Primer Sequence: 5'-3'** |
| **Forward PTEN** | **TTGTGGTCTGCCAGCTAAA** |
| **Reverse PTEN** | **CGCTCATACTGCAAATGCT** |
| **Forward β-catenin** | **CTGCTGTTTTGTTCCGAATGTC** |
| **Reverse β-catenin** | **CCATTGGCTCTGTTCTGAAGAGA** |
| **Forward c-Jun** | **GCCTCAGACAGTGCCCGAGAT** |
| **Reverse c-Jun** | **GTTTAAGCTGTGCCACCTGTTCC** |
| **Forward β-actin** | **ATGTTGAGACCTTCAACACC** |
| **Reverse β-actin** | **AGGTAGTCAGTCAGGTCCCGGCC** |
| **Forward miR-221** | **ACACTCCAGCTGGGAGCTACATTGTCTGCTGG** |
| **Reverse miR-221** | **CTCAACTGGTGTCGTGGA** |
| **Forward U6** | **CTCGCTTCGGCAGCACA** |
| **Reverse U6** | **AACGCTTCACGAATTTGCGT** |
|  |  |
