# Supplementary material for: miR-221 Promotes Epithelial-Mesenchymal Transition through Targeting PTEN and Forms a Positive Feedback Loop with β-catenin/c-Jun Signaling Pathway in Extra-Hepatic Cholangiocarcinoma
Source: PLoS One. 2015 Oct 26;10(10):e0141168. doi: 10.1371/journal.pone.0141168 (PMC4621024; doi:10.1371/journal.pone.0141168)
Supplement: S2 Table — (DOC) [file pone.0141168.s002.doc]

| **Antibodies used for Western Blot** | | | |  | |  |
| --- | --- | --- | --- | --- | --- | --- |
| **Name of Antibody** | **Manufacturer** | **Species** | **Dilution** | |  | |
| **PTEN** | **sc-7974 (Santa Cruz Biotechnology, USA)** | **Mouse** | **1:500** | |  | |
| **β-catenin** | **sc-59737 (Santa Cruz Biotechnology, USA)** | **Mouse** | **1:200** | |  | |
| **c-Jun** | **sc-376488 (Santa Cruz Biotechnology, USA)** | **Mouse** | **1:500** | |  | |
| **MMP-2** | **Sc-53630 (Santa Cruz Biotechnology, USA)** | **Mouse** | **1:500** | |  | |
| **E-cadherin** | **sc-59778 (Santa Cruz Biotechnology, USA)** | **Mouse** | **1:200** | |  | |
| **N-cadherin** | **sc-59987 (Santa Cruz Biotechnology, USA)** | **Mouse** | **1:500** | |  | |
| **β-actin** | **sc-69879 (Santa Cruz Biotechnology, USA)** | **Mouse** | **1:1000** | |  | |
